# Live birth rate after human chorionic gonadotropin priming in vitro maturation in women with polycystic ovary syndrome

**DOI:** 10.1186/s13048-018-0445-5

**Published:** 2018-08-27

**Authors:** V. N. A. Ho, T. D. Pham, A. H. Le, T. M. Ho, L. N. Vuong

**Affiliations:** 1 IVFMD, My Duc Hospital, Ho Chi Minh City, Vietnam; 2 Department of Obstetrics and Gynecology, University of Medicine and Pharmacy at Ho Chi Minh City, Ho Chi Minh City, Vietnam

**Keywords:** In vitro maturation, In vitro fertilization, Polycystic ovary syndrome, Cumulative live birth rate, Pregnancy outcomes

## Abstract

**Background:**

In vitro maturation (IVM) has some advantages over conventional in vitro fertilization (IVF), particularly in polycystic ovary syndrome (PCOS) where the risk of ovarian hyperstimulation is high. We studied the live birth rate in a large series of PCOS women undergoing human chorionic gonadotropin (hCG)-priming IVM.

**Methods:**

This retrospective study included women with PCOS aged 18–42 years undergoing IVM with hCG priming. We reported live birth rate after the first embryo transfer and cumulative live birth rate from embryos obtained in the IVM cycle. We also performed logistic regression to assess which factors predicted number of oocytes and live birth.

**Results:**

We included 921 women (age 28.9±3.5 years, body mass index 21.8±3.1 kg/m^2^, infertility duration 3.7±2.6 years, 81% primary infertility, 88% first IVF attempt, 94% ovulation induction failure). Live birth rate after the first embryo transfer was 31.7%, with a cumulative live birth rate from the cycle of 33.7%. High anti-Müllerian hormone levels predicted a high number of oocytes and a high oocyte maturation rate while the opposite was the case when luteinizing hormone levels were high.

**Conclusions:**

In women with PCOS, hCG priming IVM was feasible and resulted in acceptable live birth rates.

## Background

Currently, in vitro fertilization (IVF) involves ovarian stimulation with supra-physiological doses of gonadotrophins in order to increase the oocyte yield, thus increasing the number of embryos. Gonadotrophins are expensive, and their use carries the risk of ovarian hyperstimulation syndrome (OHSS), particular in women with polycystic ovary syndrome (PCOS) women [1, 2].

In vitro maturation (IVM) involves collection of immature oocytes that are cultured in vitro until they reach the metaphase II (MII) stage prior to insemination and does not need controlled ovarian hyperstimulation (COH). Human IVM was first reported in 1991 in an unstimulated donor cycle [3], while the first live birth after IVM in a woman with PCOS was reported in 1994 [4]. Although there were initially some concerns about IVM in terms of effectiveness and the risk of genetic abnormalities [5–8], recent studies have reported pregnancy rates after IVM comparable to IVF [9]. In addition, the risk of congenital abnormalities in IVM offspring does not appear to be increased [10–12].

The advantages of IVM over conventional IVF include lower cost (because it does not need ovarian hyperstimulation), the absence of OHSS and less inconvenience for women [13–17]. Absence of OHSS is particularly relevant for women with PCOS who are at high risk of OHSS during ovarian hyperstimulation during conventional IVF, but also have an increased risk of ovarian torsion and thromboembolism associated with high estradiol levels [1, 2, 18–20]. Therefore, IVM is a useful technique for women with PCOS [21]. Although other assisted reproductive medicine strategies, such as use of gonadotropin-releasing hormone agonist (GnRHa) trigger and freeze-only cycles, have reduced the rate of OHSS in high-risk women [22–24], some cases of severe OHSS have still been reported when these strategies have been employed [25–27]. This is particularly relevant in women with PCOS undergoing IVF who are at high risk of OHSS.

Initial data suggested that outcomes after IVM in women with PCOS are at least as good as those in women without PCOS, but the total number of PCOS women treated with IVM is relatively small [28]. In addition, although there are some published data on clinical and ongoing pregnancy rates after IVM in PCOS women [21, 29–35], only three studies involving PCOS women have reported live birth rate [9, 30, 36]. Here, we report the live birth rate in PCOS women undergoing IVM, including predictors of live birth, number of oocytes retrieved and maturation rate after IVM.

## Methods

### Study design

This retrospective study was performed in My Duc hospital, a large private IVF center in Ho Chi Minh City, Vietnam. The study was carried out in accordance with the Declaration of Helsinki, and all patient information was handled confidentially. The confidential use of patient information for research purposes is exempt from ethical approval under Vietnamese law.

### Subjects

To be eligible, women had to have PCOS diagnosed according to the Rotterdam criteria [37], be aged 18–42 years, and were undergoing IVM. Exclusion criteria included uterine abnormalities, and donor or preimplantation genetic screening/diagnosis cycles.

### Stimulation, monitoring and oocyte pick-up

The same ovarian stimulation protocol was used in all patients. Oral contraceptive pills were given for two weeks to induce bleeding. Women received injections of follicle-stimulating hormone (FSH; Puregon^®^, Merck Sharpe & Dohme) 100 IU/day on cycle days 3, 4 and 5. Ultrasound was performed on cycle day 5 to document follicular size and determine endometrial thickness. Women were given an injection of hCG 10,000 IU on cycle day 6 (independent of follicle size), and oocyte pick-up (OPU) was scheduled 36 h thereafter.

### In vitro maturation protocol

After OPU, all oocytes were placed in pre-maturation medium for 2 h. They were then transferred to maturation medium (Origio, Denmark) and cultured for 20 h, after which mature oocytes underwent intra-cytoplasmic sperm injection (ICSI). In the remaining oocytes, maturation was re-checked after another 4 h, with ICSI performed in any additional mature oocytes.

### Embryo transfer

Fresh embryo transfer (ET) was performed 3 days (day 2, 4-cell embryos) or 4 days (day 3, 8-cell embryos) after OPU based on patient preference. Any additional embryos were frozen on the day of scheduled embryo transfer. When successful pregnancy did not occur, a frozen ET was planned. In frozen ET cycles, the endometrium was prepared using oral estradiol valerate 8 mg/day starting from the second or third day of the menstrual cycle. Endometrial thickness was monitored from day 6 onwards, and vaginal progesterone 800 mg/day was started when endometrial thickness reached ≥8 mm. A maximum of three embryos were thawed on the day of ET, two or three days after the start of progesterone depending on the stage of embryo freezing. Two hours after thawing, surviving embryos were transferred into the uterus. The number of embryos transferred was dependent on the number available and patient preference. In fresh and frozen cycles, a serum beta hCG test was performed 2 weeks after ET. Luteal support with estradiol 4 mg/day and vaginal progesterone 800 mg/day was continued up to at least 7 weeks of gestation.

### Outcomes

We studied the live birth (birth of at least one newborn after 24 weeks’ gestation that exhibits any sign of life) rate after the first ET. We also report the number of oocytes retrieved, maturation rate, fertilization rate, OHSS rate, implantation rate, positive pregnancy test (serum hCG level > 5 mIU/mL), clinical pregnancy (at least one gestational sac on ultrasound at 7 weeks), ongoing pregnancy (pregnancy continuing past 20 weeks’ gestation), ectopic pregnancy (presence of a gestational sac outside the uterine cavity shown on sonography or laparoscopy) and miscarriage (pregnancy loss at < 12 weeks and from 12 to 24 weeks). In case of ongoing pregnancy, we reported preterm delivery, birth weight (grams), and any congenital anomaly. We also report median time to achieve live birth, and the cumulative live birth rate at 12 months after the IVM cycle.

### Statistical analysis

Outcomes after the first ET are reported using descriptive statistics. We constructed Kaplan-Meier curves to estimate cumulative live birth rates. Univariate and multivariate logistic regression analyses were performed to identify predictive variables of live birth after the first ET. Univariate and multivariate linear regression analyses were also performed to identify predictive variables for the number of oocytes retrieved and for the maturation rate. All variables with *p* values of < 0.25 in the univariate analysis were included in the multivariate analyses to identify independent predictors of live birth after IVM. All analyses were performed using the R statistical package (R version 3.3.3).

## Results

### Participants

**Fig. 1:**
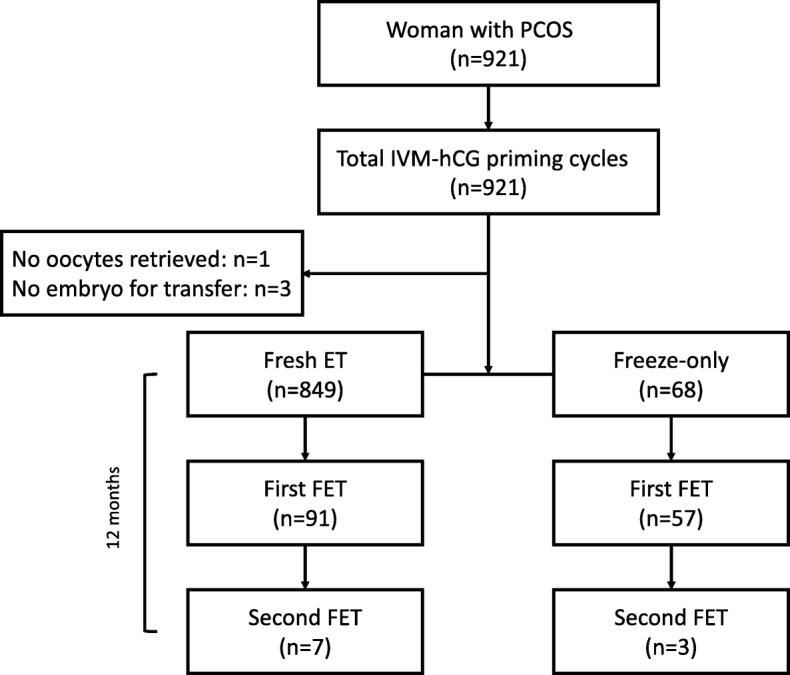
Flow diagram showing number of women undergoing embryo transfer. ET, embryo transfer; FET, frozen embryo transfer; hCG, human chorionic gonadotropin; PCOS, polycystic ovary syndrome

A total of 921 PCOS women underwent IVM between April 2014 and October 2016 (mean age 28.9 ± 3.5 years, body mass index [BMI] 21.8±3.1 kg/m^2^, infertility duration 3.7±2.6 years) (Fig. 1, Table 1), The majority of women had primary infertility, were undergoing their first IVF attempt, and had ovulation induction failure as the indication for IVM (Table 1).

**Table 1:** Demographic and clinical characteristics of participants at baseline

Characteristics	Participants (*n* = 921)
Age, years	28.9 ± 3.5
Body mass index, kg/m^2^	21.8 ± 3.1
Anti-Müllerian hormone, ng/mL	12.3 ± 3.6
Antral follicle count, *n*	34.3 ± 18.3
Follicle-stimulating hormone^a^, IU/mL	8.5 ± 1.9
Progesterone^a^, ng/mL	0.7 ± 1.8
Luteinizing hormone^a^, IU/L	14.3 ± 6.9
Free testosterone index^a^	5.2 ± 4.7
Menstrual cycles, *n* (%)	
Amenorrhea	52 (5.6)
Oligomenorrhea	850 (92.3)
Regular	19 (2.0)
Polycystic ovaries on ultrasound, *n* (%)	
Both ovaries	777 (84.4)
One ovary	144 (15.6)
Duration of infertility, years	3.7 ± 2.6
Type of infertility, *n* (%)	
Primary	747 (81.1)
Secondary	174 (18.9)
Number of IVF attempts, *n*, (%)	
1	814 (88.4)
2	80 (8.7)
3	27 (2.9)
Indications for IVM, *n* (%)	
Tubal factors	18 (2.0)
Male factors	13 (1.4)
Ovulation induction failure	862 (93.6)
Previous OHSS with COH	18 (2.0)
Other	10 (1.1)

Values are mean ± standard deviation, or number of women (%)

*COH*, controlled ovarian hyperstimulation, *IVF* in vitro fertilization, *IVM* in vitro maturation, *OHSS* ovarian hyperstimulation syndrome

^a^Hormone levels were assessed at the first physician visit, on the second day of progestogen-induced bleeding

### Controlled ovarian stimulation

The mean number of oocytes retrieved per woman was almost 15, nearly all embryos were Day 2, and maturation and fertilization rates were both approaching 70% (Table 2). The proportion of oocytes that were mature at the time of collection was about 7% (Table 2). No cases of OHSS were recorded.

**Table 2:** Laboratory outcomes

	Participants (*n* = 921)
Number of oocytes retrieved^a^	14.9 ± 8.9
Number of oocytes matured at collection	1.1 ± 2.3
Proportion of mature oocytes at collection, %	7.2 ± 14.5
Maturation rate, %	71.2 ± 14.5
Fertilization rate, %	68.2 ± 22.1
Number of embryos^b^	6.0 ± 3.6
Stage of embryos, *n* (%)	
Day 2	908 (98.6)
Day 3	9 (1.0)
Number of frozen embryos	1.5 ± 2.2

Values are mean ± standard deviation, or number (%)

^a^One woman without oocytes; ^b^Three women without embryos

### Treatment outcomes

**Fig. 2:**
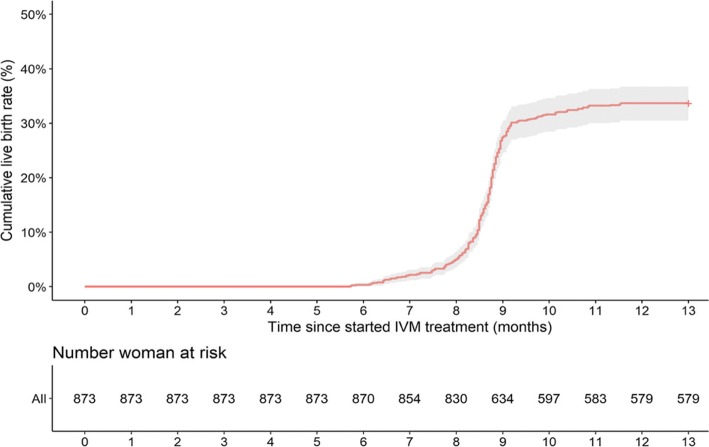
Cumulative live birth rate over 12 months after one IVM cycle

The live birth rate after the first IVM cycle ET was 31.7% (32.0% after fresh ET and 28.0% after frozen ET). The clinical pregnancy rate was higher (43.5%) but 10.3% of all pregnancies ended with miscarriage (Table 3). Additional embryo transfers were performed in 94 women. In the 12 months after the IVM cycle, 974 ETs were performed (fresh and/or frozen) in 873 women. The cumulative live birth rate was 33.7% (Fig. 2, Table 3). The median time to achieve live birth was 8.7 months. No babies were born with congenital abnormalities.

**Table 3:** Pregnancy outcomes

	Overall^a^ (*n* = 873)	Type of first embryo transfer	
		Fresh (*n* = 823)	Frozen (*n* = 50)
Number of embryos transferred	2.5 ± 0.6	2.5 ± 0.6	2.5 ± 0.6
After first embryo transfer			
Live birth, *n* (%)	277 (31.7)	263 (32.0)	14 (28.0)
Singleton	175/277 (63.2)	165/263 (62.7)	10/14 (71.4)
Twins	102/277 (36.8)	98/263 (37.3)	4/14 (28.6)
Positive beta hCG, *n* (%)	438 (50.2)	417 (50.7)	21 (42.0)
Clinical pregnancy, *n* (%)	380 (43.5)	361 (43.9)	19 (38.0)
Ongoing pregnancy, *n* (%)	286 (32.8)	272 (33.0)	14 (28.0)
Implantation, %	25.1	25.3	20.7
Ectopic pregnancy, *n* (%)	12 (1.4)	11 (1.3)	1 (2.0)
Miscarriage (before 12 weeks), *n* (%)	81 (9.3)	76 (9.2)	5 (10.0)
Miscarriage (between 12 and 24 weeks), *n* (%)	9 (1.0)	9 (1.1)	0 (0.0)
Birth weight, g			
Singleton	2980.7 ± 733.4	2951.3 ± 737.2	3433.3 ± 625.0
Twins	2325.8 ± 545.5	2302.1 ± 538.1	2412.5 ± 513.2
At 12 months			
Total number of embryo transfers, *n* (%)^b^			
1	779 (89.2)		
2	87 (10.0)		
3	7 (0.8)		
Live birth, *n* (%)	294 (33.7)		
Singleton	192/294 (65.3)		
Twins	102/294 (34.7)		
Positive beta hCG, *n* (%)	508 (58.2)		
Clinical pregnancy, *n* (%)	440 (50.4)		
Ongoing pregnancy, *n* (%)	326 (37.3)		
Implantation, %	25.6		
Ectopic pregnancy, *n* (%)	14 (1.6)		
Miscarriage (before 12 weeks), *n* (%)	102 (11.6)		
Miscarriage (between 12 and 24 weeks), *n* (%)	9 (1.0)		
Birth weight, g			
Singleton	2969.9 ± 737.3		
Twins	2307.2 ± 536.0		

Values are mean ± standard deviation, or number (%)

^a^Thirty-three women were lost to follow-up after the first transfer, 11 did not undergo embryo transfer, one woman had no oocytes retrieved, and three women had no embryos to transfer

*hCG* human chorionic gonadotropin

^b^A total of 1007 transfers were performed, but no follow-up data were available for 33, meaning that data from 974 transfers are reported

### Predictors of live birth

While a number of factors were significant predictors of live birth in the univariate analysis (including age, BMI, duration of infertility, and number of IVF attempts), only the number of embryos available for transfer and the number of embryos transferred were significant independent predictors of live birth after IVM on multivariate logistic regression analysis (Table 4). After transfer of a single embryo, the live birth rate was 80% lower than when two embryos were transferred (*p* = 0.032) but transferring three embryos did not significantly improve the live birth rate compared with transferring two embryos.

**Table 4:** Factors influencing the live birth rate after first embryo transfer after in vitro maturation

Predictive factors	Live births (*n* = 277)	No live births (*n* = 596)	Univariate analysis		Multivariate analysis	
			Odds ratio (95% CI)	*p*-value	Odds ratio (95% CI)	*p*-value
Pretreatment factors (*n* = 873)^b^						
Age, years^a^	28.1 ± 3.5	29.1 ± 3.5	0.94 (0.90, 0.98)	0.004	0.97 (0.93, 1.02)	0.254
Body mass index, kg/m^2a^	21.5 ± 2.8	22.0 ± 3.2	0.94 (0.90, 0.99)	0.019	0.95 (0.90, 1.00)	0.06
Anti-Müllerian hormone, ng/mL^a^	12.5 ± 3.6	12.2 ± 3.6	1.02 (0.98, 1.06)	0.304	–	–
Antral follicle count – *n*	34.3±17.9	34.4±18.5	1.00 (0.99, 1.01)	0.96	–	–
Follicle-stimulating hormone, IU/mL	8.6 ± 1.9	8.5 ± 1.8	1.03 (0.95, 1.11)	0.524	–	–
Progesterone, ng/mL	0.8 ± 1.9	0.7 ± 1.8	1.01 (0.93, 1.09)	0.761	–	–
Luteinizing hormone, IU/L	14.3 ± 6.9	13.5 ± 6.7	1.02 (1.00, 1.04)	0.105		
Free testosterone index	4.5 ± 5.6	4.6 ± 5.2	1.00 (0.97, 1.02)	0.786	–	–
Menstrual cycles, *n* (%)						
Amenorrhea	15 (5.4)	33 (5.5)	Ref		–	–
Oligomenorrhea	256 (92.4)	551 (92.4)	1.02 (0.56, 1.97)	0.946	–	–
Regular	6 (2.2)	12 (2.0)	1.10 (0.33, 3.43)	0.871	–	–
Polycystic ovaries on ultrasound, *n* (%)						
Both ovaries	228 (82.3)	488 (81.9)	Ref		–	–
One ovary	49 (17.7)	108 (18.1)	0.97 (0.67, 1.41)	0.88	–	–
Duration of infertility, years^a^	3.3 ± 2.3	3.8 ± 2.7	0.92 (0.86, 0.98)	0.011	0.95 (0.89, 1.02)	0.161
Type of infertility, *n* (%)						
Primary	229 (82.7)	479 (80.4)	Ref		–	–
Secondary	48 (17.3)	117 (19.6)	0.86 (0.59, 1.24)	0.419	–	–
Number of IVF attempts, *n* (%)						
1	259 (93.5)	514 (86.2)	Ref		–	–
2	11 (4.0)	64 (10.7)	0.34 (0.17, 0.63)	0.001	0.60 (0.32, 1.09)	0.105^c^
3	7 (2.5)	18 (3.0)	0.77 (0.30, 1.80)	0.566	–	–
Indications for IVM, *n* (%)						
Tubal factors	3 (1.1)	14 (2.3)	Ref		–	–
Male factors	2 (0.7)	11 (1.8)	0.85 (0.10, 6.01)	0.869	–	–
Ovulation induction failure	267 (96.4)	550 (92.3)	2.27 (0.73, 9.89)	0.202	1.92 (0.92, 4.38)	0.097
Previous OHSS with COH	3 (1.1)	14 (2.3)	1.00 (0.16, 6.24)	0.99	–	–
Others	2 (0.7)	7 (1.2)	1.33 (0.15, 9.99)	0.779	–	–
Treatment factors (*n* = 873)^b^						
Number of oocytes retrieved^a^	15.6 ± 8.4	14.7 ± 9.1	1.01 (0.99, 1.03)	0.182	0.98 (0.95, 1.01)	0.286
Number of mature oocytes after IVM^a^	9.1 ± 5.0	8.5 ± 5.3	1.02 (0.99, 1.05)	0.109	0.97 (0.92, 1.02)	0.212
Number of embryos available for transfer^a^	6.8 ± 3.6	5.7 ± 3.6	1.09 (1.05, 1.13)	< 0.001	1.14 (1.07, 1.22)	< 0.001
Mean number of embryos transferred^a^	2.6 ± 0.5	2.5 ± 0.6	1.55 (1.20, 2.01);	0.001		
Number of embryos transferred, *n* (%)						
1 embryo transferred	2 (0.7)	34 (5.7)	0.14 (0.02, 0.47)	0.008	0.20 (0.03, 0.69)	0.032
2 embryos transferred	104 (37.5)	247 (41.4)	Ref	–	Ref	–
3 embryos transferred	171 (61.7)	315 (52.9)	1.29 (0.96, 1.73)	0.092	1.25 (0.92, 1.69)	0.152
Embryo transfer method, *n* (%)						
Fresh	263 (94.9)	560 (94.0)	Ref		–	–
Freeze-only	14 (5.1)	36 (6.0)	0.83 (0.43, 1.53)	0.56	–	–

*CI* confidence interval, *COH* controlled ovarian hyperstimulation, *IVF* in vitro fertilization, *IVM* in vitro maturation, *OHSS* ovarian hyperstimulation syndrome

^a^Parameters analyzed as continuous variables; odds ratio values are per unit increase

^b^Thirty-three women were lost to follow-up after the first transfer, and 11 did not undergo embryo transfer, one woman had no oocytes retrieved, and three women had no embryos to transfer

### Predictors of number of oocytes and maturation rate

Factors significantly correlated with the number of oocytes on multivariate analysis were BMI, anti-Müllerian hormone (AMH) level, luteinizing hormone (LH) level, number of previous IVF attempts, and ovulation induction failure as the indication for IVM (Table 6). When BMI, AMH and the number of previous IVF attempts were higher, the number of oocytes retrieved was higher (*p* < 0.001, *p* < 0.001 and *p* = 0.006, respectively) (Table 5). Conversely, as LH increased the number of oocytes decreased (*p* = 0.03) (Table 5). PCOS women with ovulation induction failure had a significantly higher number of oocytes than those with tubal factor indications for IVM (*p* = 0.004) (Table 5). BMI (*p* < 0.001), AMH (*p* = 0.01) and number of previous IVF attempts (*p* = 0.008) were also significant predictors of the maturation rate on multivariate analysis, with similar relationships to those for oocyte number (Table 5).

**Table 5:** Factors influencing oocyte maturation (*n* = 920) and the number of oocytes retrieved (*n* = 921) after in vitro maturation

	Oocyte maturation				Oocytes retrieved			
	Univariate analysis		Multivariate analysis		Univariate analysis		Multivariate analysis	
	β	*p*-value	β	*p*-value	β	*p*-value	β	*p*-value
Age, years	0.001	0.99	–	–	0.11	0.19	−0.08	0.31
Body mass index, kg/m^2^	−0.1	0.70	–	–	0.845	< 0.001	0.85	< 0.001
Anti-Müllerian hormone, ng/mL	0.31	0.15	0.32	< 0.001	0.28	0.001	0.36	< 0.001
Antral follicle count, *n*	0.07	0.27			0.002	0.88		
Follicle stimulating hormone, IU/mL	0.26	0.53	–	–	0.14	0.39	–	–
Progesterone, ng/mL	0.35	0.41	–	–	−0.17	0.31	–	–
Luteinizing hormone, IU/L	0.48	< 0.001	−0.11	0.01	−0.09	0.04	−0.09	0.03
Free testosterone index	0.44	0.002	−0.05	0.37	−0.03	0.62	–	–
Menstrual cycles, *n* (%)								
Amenorrhea	Ref	Ref	–	–	Ref	Ref	–	–
Oligomenorrhea	−6.68	0.05	−0.82	0.53	−0.82	0.54	–	–
Regular	−5.74	0.36	–	–	−3.96	0.11	−3.97	0.08
Polycystic ovaries on ultrasound, *n* (%)								
Both ovaries	Ref	Ref	–	–	Ref	Ref	–	–
One ovary	0.92	0.65	–	–	−1.06	0.26	–	–
Duration of infertility, years	0.21	0.48	–	–	−0.02	0.86	–	–
Type of infertility, *n* (%)								
Primary	Ref	Ref	Ref	Ref	Ref	Ref	Ref	Ref
Secondary	−5.79	0.003	1.15	0.13	1.38	0.07	1.17	0.11
Number of IVF attempts, *n* (%)								
1	Ref	Ref	Ref	Ref	Ref	Ref	Ref	Ref
2	−7.65	0.005	1.33	0.21	1.63	0.13	1.74	0.11
3	−5.77	0.21	4.71	0.008	4.51	0.01	4.84	0.006
Indications for IVM, *n* (%)								
Tubal factors	Ref	Ref	–	–	Ref	Ref	–	–
Male factors	−0.60	0.94	–	–	−2.24	0.49	–	–
Ovulation induction failure	−1.19	0.83	–	–	3.92	0.07	4.05	0.004
Previous OHSS with COH	−6.72	0.38	–	–	5.12	0.09	2.22	0.38
Others	−0.90	0.80	–	–	− 0.90	0.80	–	–

*COH* controlled ovarian hyperstimulation, *IVF* in vitro fertilization, *IVM* in vitro maturation, *OHSS* ovarian hyperstimulation syndrome

## Discussion

The results of this retrospective analysis show that acceptable live birth rates can be achieved in women with PCOS after IVM with hCG priming. The live birth rate after IVM was close to that with conventional IVF. There are a number of possible reasons for this. Firstly, we have been doing IVM for nearly 12 years so have a good level of experience and competency with this technique. Secondly, the IVF rate at our center is slightly lower than that at other centers because we transfer day 3 embryos rather than blastocysts.

A limited number of previous studies have reported the live birth rate after IVM in women with polycystic ovary morphology or PCOS. In one of these, the cumulative live birth rate per oocyte collected (80 cycles of IVM in 56 women) was 41.3% [9]. In another study, the live birth rate per oocyte collected was also slightly higher, at 42.4% [30]. In our study, the live birth rate after the first embryo transfer, and cumulative live birth rate for one IVM cycle were lower (31.7% and 33.7%, respectively). However, direct comparison between ours and previous studies is difficult because both the previous studies did not use hCG priming (which facilitates fresh embryo transfer), and transferred a single blastocyst using fresh [30] or used fresh and frozen ET [9]. Fresh transfer of day 2 or day 3 embryos rather than blastocysts could be one potential explanation for the difference in live birth rate between the current study and existing data.

The live birth rate in our study was comparable to the 34.6% reported with fresh transfer of one or two day 3 embryos after IVM and ICSI in PCOS women from Canada and Turkey [36], and nearly twice that reported after frozen transfer of one or two day 3 embryos in women with PCOS in a European retrospective study (16.2%) [38]. Given that these studies and ours transferred day 3 embryos after IVM, the reasons underlying the lower live birth rate in the European study are not clear. Importantly, the live birth rate achieved after IVM based on the results of this analysis was comparable to that achieved with conventional IVF in similar women from Vietnam [39, 40].

We identified the number of embryos available for transfer and the number of embryos transferred as significant independent predictors of live birth after IVM on multivariate logistic regression analysis, consistent with existing data [36, 41]. In our study, up to three embryos were transferred. The transfer of three embryos in some cases was based on earlier data suggesting that IVM embryos are not usually as good as IVF embryos. However, data from this analysis suggested that the transfer of more than two embryos did not result in significantly higher live birth rate. Data from a small study suggest that acceptable live birth rates can be achieved after IVM with single blastocyst transfer [9]. Although we found that additional variables were statistically significant predictors of live birth in univariate analyses, these did not persist in the multivariable model, possibly because the number of embryos and the number of embryos transferred are such strong and direct predictors of pregnancy and live birth.

Collection of a good number of oocytes is the first step in the IVM procedure and their maturation is essential so that there are a good number of embryos to transfer. To facilitate selection of suitable candidates prior to starting IVM, pretreatment factors associated with relevant procedural metrics are needed. This was the rationale for our analysis of predictive factors for number of oocytes and maturation rate.

Associations between serum AMH and/or AFC and live birth have been reported previously [29, 42], but these variables were not significant independent predictors of live birth in our analysis. However, we did identify significant associations between AMH and both the number of oocytes and the oocyte maturation rate. Conversely, these parameters decreased significantly when levels of LH were high. The reason for this is unclear given that a positive association between LH and AMH serum levels, independent of serum androgen levels, has been reported previously [43]. Baseline LH in our study (14.3 IU/L) was higher than that in patients from a previous similar study (9.1 IU/L) [29], but the importance of this remains to be determined. In a previous model to identify PCOS women suitable for IVM, the presence of at least eight cumulus oocyte complexes was identified as being associated with a higher number of good quality embryos and a better ongoing pregnancy rate [29]. Duration of infertility has previously been reported as a significant predictor of live birth after IVM [36]. Although infertility duration was a significant predictor of live birth on univariate analysis, this association did not persist in the multivariate model. However, we did identify significant independent associations between the number of previous IVF attempts and both oocyte number and maturation rate.

BMI was not a significant predictor of live birth after IVM but was significantly associated with the number of oocytes retrieved during the procedure. This is in contrast to data from a retrospective cohort study that failed to find any association between BMI and the number and quality of oocytes in PCOS women undergoing IVM [44]. The influence of BMI on IVM is an important area for future study, particularly with respect to potential ethnic differences, because Asian women with PCOS are usually lean and have a lower BMI than Western PCOS populations [45].

In 2013, an American Society of Reproductive Medicine (ASRM) committee described IVM as “an experimental procedure” that should only be performed “in specialized centers for carefully selected patients”, and that candidates for this approach to infertility treatment include those at risk for OHSS and women with PCOS [46]. Our study was conducted at a specialist center and in the target groups described and showed the potential for live birth rates after IVM to approach those achieved with traditional IVF. There is also much debate about how IVM is defined, and there is not yet any consensus about a standard protocol for IVM. The ASRM committee recently defined IVM as “maturation in culture of immature oocytes after their recovery from follicles which may or may not have been exposed to FSH but were not exposed to either LH or hCG prior to retrieval to induce meiotic resumption.” Use of hCG priming does not fulfil these criteria. However, this approach has been used by some centers because it was reported to improve oocyte maturation, development potential and outcomes [47–50]. However, data from a randomized controlled trial show that this was not associated with any difference between hCG-primed and non-primed IVM cycles in terms of subsequent embryo developmental competence [51]. Furthermore, despite an overall low quality of available evidence, a Cochrane review concluded that hCG-priming had no effect on pregnancy, miscarriage or live birth rates with IVM using day 2–3 embryos, although well-designed randomized clinical trials in this area are required [52].

The hCG priming approach was the most common protocol at our institution over the period from which study data were analyzed. One of the advantages of using hCG priming is that fresh embryo transfer is possible due to the direct effects of hCG on the endometrium [53]. A disadvantage of hCG priming is that oocytes are at different levels of maturity at OPU, meaning that there is a need to do multiple checks for maturation and then perform several rounds of ICSI whenever mature oocytes are detected [54], both of which increase the laboratory workload and procedural costs. There is a need for more randomized controlled trials comparing hCG-priming and non-hCG-priming IVM to provide reliable data on the comparative effectiveness, complications, time to pregnancy, neonatal health, safety, costs and cost effectiveness of the two approaches. Additional research is required to define the optimal protocol for IVM. Whichever protocol is used, IVM is increasingly being associated with acceptable pregnancy outcomes and provides a promising alternative ART approach for specific groups of women that is efficient, convenient, safe and more financially accessible for many couples.

The retrospective design of this study was the most important limitation; however a large number of patients were included. In addition, pregnancy-related complications (e.g. pre-eclampsia, diabetes mellitus) were not documented. The age range of included patients was wide, but there were few patients at either end of the range, with the majority of patients being aged between 23 and 35 years. Our findings are only applicable to the specific approach taken to IVM in this study. The transfer of day 2 embryos is not commonly practiced in many IVF centers, however, there is actually no standard protocol for IVM and we are currently working to find out the optimal protocol for doing IVM. Since the period over which data were collected, the IVM protocol used at our center has changed, and now uses a non-hCG approach with transfer of two day 3 embryos, which is closer to modern ART practice. Although differences in IVM protocols are common, making comparison between studies and consensus difficult, IVM should continue to be studied because it is a promising ART technique. A key strength of this study is the large sample size, although the number of women in some subgroups used to analyze predictors of live birth was small. To the best of our knowledge, this is the largest study of IVM in PCOS to date and one of only a few to report live birth rate and cumulative live birth rate. Better understanding of IVM will improve its application in clinical practice and help with selecting women more likely to have a positive outcome. Improvements in IVM techniques are also contributing to better outcomes with this important ART technique [55]. In addition, because it can be performed rapidly without hormonal stimulation, IVM is a promising technique for preserving fertility in women with cancer who require gonadotoxic antineoplastic therapy [56].

## Conclusions

The live birth rate after IVM in women with PCOS was acceptable and similar to that after IVF. IVM is a convenient option in these women and may be a feasible and effective alternative to IVF. Women with a high AMH and low LH at baseline appear to be most suitable for IVM, and transfer of two embryos gives a good result in terms of live birth rate.
